# Effect of multiple binge alcohol on diet-induced liver injury in a mouse model of obesity

**DOI:** 10.1038/nutd.2015.4

**Published:** 2015-04-27

**Authors:** A M P Duly, B Alani, E Y-W Huang, C Yee, P S Haber, S V McLennan, D Seth

**Affiliations:** 1 Centenary Institute of Cancer Medicine and Cell Biology, Camperdown, NSW, Australia; 2 Department of Endocrinology, Royal Prince Alfred Hospital, Camperdown, NSW, Australia; 3 Sydney Medical School, University of Sydney, Sydney, NSW, Australia; 4 Drug Health Services, Royal Prince Alfred Hospital, Camperdown, NSW, Australia

## Abstract

**Background:**

Alcoholic liver disease (ALD) and non-alcoholic fatty liver disease (NAFLD) are highly prevalent liver diseases that may coexist and contribute significantly to liver disease-related mortality. Obesity is a common underlying risk factor for both disorders. There has been little research investigating the combined effects of high fat diet (HFD) and alcohol. Current mouse models of alcohol- or fat-rich diet alone do not lead to severe liver injury. There is a need to develop animal models recapitulating human settings of drinking and diet to study the mechanisms of liver injury progression.

**Methods:**

C57BL6 male mice were fed either chow or HFD *ad libitum* for 12 weeks. A sub-set of mice from each group were also given alcohol (2 g kg^−^^1^ body weight) twice a week via intra-gastric lavage. Animals were monitored progressively for weight gain and blood and livers were harvested at termination. The extent of liver injury was examined by histopathology as well as by liver and serum biochemistry. The expression of lipid metabolism, inflammation and fibrogenesis-related molecules was examined by quantitative reverse transcription PCR (Q-PCR) and immunofluorescence staining.

**Results:**

HFD significantly increased total body weight, triglyceride and cholesterol, whereas alcohol increased liver weight. Alcohol+HFD in combination produced maximum hepatic steatosis, increased micro- and macro-vesicular lipid droplets, increased *de novo* lipogenesis (steroid response-element binding protein 1 (SREBP-1) and stearoyl-CoA desaturase-1 (SCD-1)) and proliferation peroxisome activated receptor alpha (PPARα), and decreased fatty acid β-oxidation (Acyl-CoA oxidase 1 (ACOX1)). Alcohol+HFD treatment also increased the inflammation (CD45+, CD68+, F4/80+ cells; tumour necrosis factor-alpha (TNF-α), F4/80 mRNAs) and fibrogenesis (vimentin+ activated stellate cells, collagen 1 (Col1) production, transforming growth factor-beta (TGF-β) and Col-1 mRNAs) in mice livers.

**Conclusions:**

We report a novel mouse model with more severe liver injury than either alcohol or HFD alone recapitulating the human setting of intermittent alcohol drinking and HFD.

## Introduction

Chronic liver disease (CLD) is one of the most prominent causes of death in the developed world.^1^ While there are many different etiologies, the prevalence of alcoholic liver disease (ALD) and non-alcoholic steatohepatitis (NASH) together account for a major proportion of liver disease burden in Australia.^2^ Alcoholic steatosis can develop in >90% of chronic excessive (>20 g day^−^^1^, female; >40 g day^−^^1^, male) drinkers, can progresses to alcoholic steatohepatitis (ASH) in 35% and to fibrosis and cirrhosis in up to 15% of chronic drinkers.^3^ ALD is associated with high morbidity and mortality, is an important contributor to the progression of hepatitis C (HCV) and is a risk factor for hepatocellular carcinoma (HCC) further increasing the burden of disease.

The disease spectrum of NAFLD resembles ALD, progressing from simple steatosis to NASH and cirrhosis. It is characterized by the deposition of hepatic fat in patients who drink <20 g (female)/<40 g (male) alcohol/day.^4^ While NAFLD is treatable with the correct diet change, progression to NASH will occur in approximately 10–20% of patients^4, 5^ who are generally obese, have aspects of the metabolic syndrome and suffer from diabetes.^6^ Recent years have shown a tremendous rise in the incidence of NASH related to increasing obesity and sedentary lifestyle.^7^ The progression to NASH mimics that seen in ASH, and NASH can also progress to cirrhosis and HCC.^6, 8^ Recent studies show that drinkers who are obese are more likely to develop cirrhosis than those within a health weight range,^9, 10^ implying the potential for an interaction in ALD and NASH, which could also be accelerated in obese drinkers.

Experimental models of alcohol and high fat diet (HFD) alone have proven difficult to induce severe injury in the liver even after several weeks of treatment.^11, 12^ For example, induction of diabetes was required to accelerate liver injury in diet-related obesity models.^12^ In alcoholic liver injury, LPS is commonly required as a ‘second hit' agent in addition to alcohol to advance steatosis to steatohepatitis. Recent model of ‘acute on chronic alcohol'^11^ removes the need for a secondary agent to induce liver injury, but there is little evidence for progression to steatohepatitis or fibrosis in this model. Murine models of alcohol and HFD have recently been reported to induce synergistic injury in the liver. However, these models had extreme regimens of alcohol administration and calorie intake, for example, daily gavage with alcohol (4 g kg^−^^1^ body weight) and 60% kcal fat diet^13^ and intragastric alcohol infusion (32 g kg^−^^1^ body weight) and up to 986 Cal kg^−^^1^ per day.^14^ In the present study, we have recapitulated in a mouse model intermittent chronic alcohol intake (2 g kg^−^^1^ body weight) and HFD (45% kcal fat) comparable to that commonly observed in the human setting of episodic heavy drinking and the prevalent fat-rich food to study the interaction between alcohol and a HFD on liver injury.

## Materials and methods

### Alcohol and HFD mouse model

Wild-type (WT) male C57BL6 mice were purchased from Animal Resource Centre (ARC) (Western Australia, Australia). Treatment commenced when mice were 6–8 weeks old and weighed approximately 20 g. The mice were fed either a normal chow diet consisting of 12% kcal fat (Chow) or HFD containing 45% kcal fat and 0.25% cholesterol for 12 weeks as described.^12^ Half the mice from the Chow and HFD group (*n*=7–8 animals per group) also received alcohol twice a week for 12 weeks (2 g kg^−^^1^ body weight as a 30% solution in saline) via gastric lavage (gavage).^15^ Control mice were given equal volumes of 100% saline. Mice were weighed before every gavage and alcohol dose was calculated for each mouse before alcohol administration (Supplementary Table 1). Mice were administered alcohol in the morning and monitored extensively (signs of distress, body coordination, eating, drinking, activity) (Supplementary Table 2), following each treatment every 30 min for 2 h, then every hour (up to 6 h total) and at 24 h to ensure full recovery. A 48- to 96-h recovery period was allowed between alcohol treatments. Mice were killed 1 week following the last treatment (that is, week 13) and serum and liver tissue samples were collected. Animal experiments were performed in accordance with Animal Ethics Committee requirements of this institution (Protocol 2011/044).

### Serum and liver analysis

Serum was analysed for alanine aminotransferase (ALT) and aspartate aminotransferase (AST) to assess liver function. Serum and liver homogenates were also analysed for triglycerides and cholesterol to assess fat content^15^ and circulating insulin.^12^ Liver tissue was used for RNA and protein expression studies.

### Immunohistochemistry and immunofluorescence

Formalin-fixed liver tissue sections were used for haematoxylin and eosin (H&E) staining. Slides were examined using a Leica DM6000B microscope (Leica Microsystems, Wetzlar, Germany) using Leica Application Suite v 4.2. Frozen liver tissues (Tissue-Tek O.C.T 4583 Sakura Finetechnical, Tokyo, Japan) were sectioned into 5 nm slices at −15 °C, fixed in 100% ethanol, air-dried and stored at −80 °C as previously described.^16^ Picrosirius red (PSR) and Oil Red O (ORO) staining was performed as described.^12, 15^ For immunofluorescence, frozen liver sections were treated with 10% paraformaldehyde for 20 min followed by permeabilisation in 0.5% Triton-X 100 (Tx-100) in phosphate-buffered saline (PBS) for 20 min. Sections were blocked using 10% normal donkey serum in PBS. Sections were stained with primary and secondary antibodies (Supplementary Table 3) diluted in 0.1% Tx-100, 10% normal donkey serum in PBS. Sections were also stained with IgG as isotype controls.

### Scoring of histopathology slides for inflammation and fibrosis

H&E slides of all animals were scored by two observers blinded for the source of the tissue for severity of inflammation (number and size of inflammatory clusters, hepatomegaly). Overall inflammation was scored as follows: 1=mild, 2=intermediate, 3=severe; cluster numbers: 1=zero, 2=1–10 clusters, 3=>10 clusters. Hepatomegaly was scored as follows: 1=mild, 2=intermediate, 3=severe. PSR-stained slides were scored similarly for degree of fibrosis: 1=mild, 2=intermediate (pericellular fibrosis), 3=strong (some bridging fibrosis). Data obtained were analysed by chi-square.

### RNA, cDNA and quantitative Q-PCR

RNA extraction and cDNA generation were performed as previously described.^15^ Quantitative reverse transcription-PCR (Q-PCR) was performed on cDNA transcribed using Superscript III (Invitrogen, Life Technologies Australia Pty Ltd, Mulgrave, Victoria, Australia) with gene-specific primers (Supplementary Table 3) and SYBR Select Master Mix (Invitrogen) as per the manufacturers' recommendation. Data are normalised to housekeeper heat-shock protein 90 alpha (cytosolic), class B member 1 (*Hsp90ab1*) mRNA levels and expressed as fold change from control.

### Statistical analysis

All experiments were repeated at least three times. Data are reported as means±s.d. unless otherwise stated. Analyses for statistical significance were performed in GraphPad Prism 6 (GraphPad Software, Inc., La Jolla, CA, USA) and Microsoft Office 2010 Excel using Student's *t*-test, one-way ANOVA with Tukey's multiple comparisons test, Chi-square analysis and Fisher's exact test as detailed in Materials and methods and/or figure and table legends as appropriate. Images represent typical experimental results.

## Results

### The combination of alcohol and HFD increased total body and liver weight

**Figure 1:**
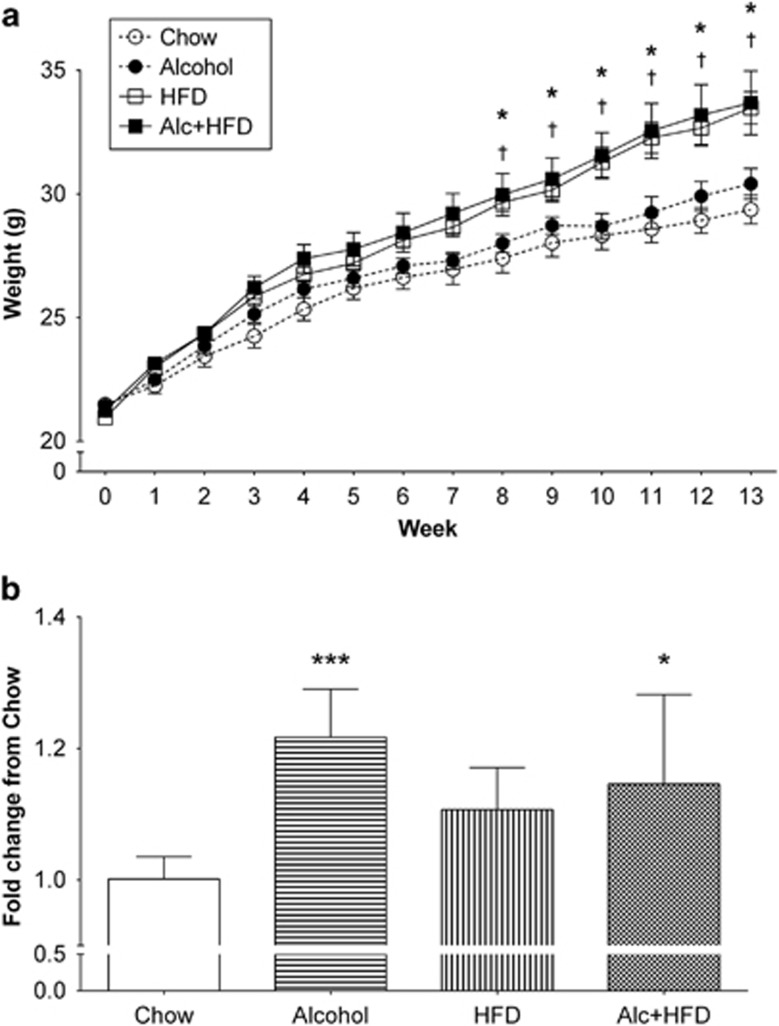
Physical characterisation of the model. (**a**) Body weight in grams (g) per week. Mice were weighed 1 week before treatment (week zero), then twice a week for 12 weeks before alcohol administration. Mice were weighed at week 13 end point, 1 week after the last alcohol dose. Mice on HFD and Alc+HFD showed maximum weight gain over the 13-week period compared with chow fed mice (Chow). Alcohol had a slight increase in weight over Chow, but did not reach significance. Data are mean±s.e.m.; significance by Student's *t*-test. (**b**) Liver-to-body weight ratio increased with Alcohol and Alc+HFD. Increase in the liver weight was observed with all treatments compared with the Chow: Alcohol 1.23-fold; HFD 1.11-fold; Alc+HFD 1.15-fold, but reached significance only with Alcohol and Alc+HFD (*n*=7–8). Data are mean±s.d.; *P<0.05, ***P<0.001 from Chow, ^†^*P*<0.05 from Alcohol (only in **a**). Alc+HFD, Alcohol+high fat diet.

HFD significantly increased average total body weight at week 7 compared with Chow and continued throughout the study (Figure 1a). Alcohol administration increased body weight in both Alcohol alone and Alcohol+HFD groups, starting at week 4 of administration but this failed to reach statistical significance. The liver-to-body weight ratio significantly (*P*<0.05) increased with alcohol alone and in combination with HFD compared with the Chow group (Figure 1b). The ratios of the weight of other organs (kidney, heart, spleen) to body weight were not altered by the treatments (Table 1). Liver transaminases (ALT, AST and AST:ALT) were not altered by any treatment but the liver ALP was increased by the HFD and Alcohol+HFD interventions compared with Chow fed animals (Table 2).

### Alcohol increased lipid accumulation and regulated lipid processing gene expression in livers of HFD-treated mice

**Figure 2:**
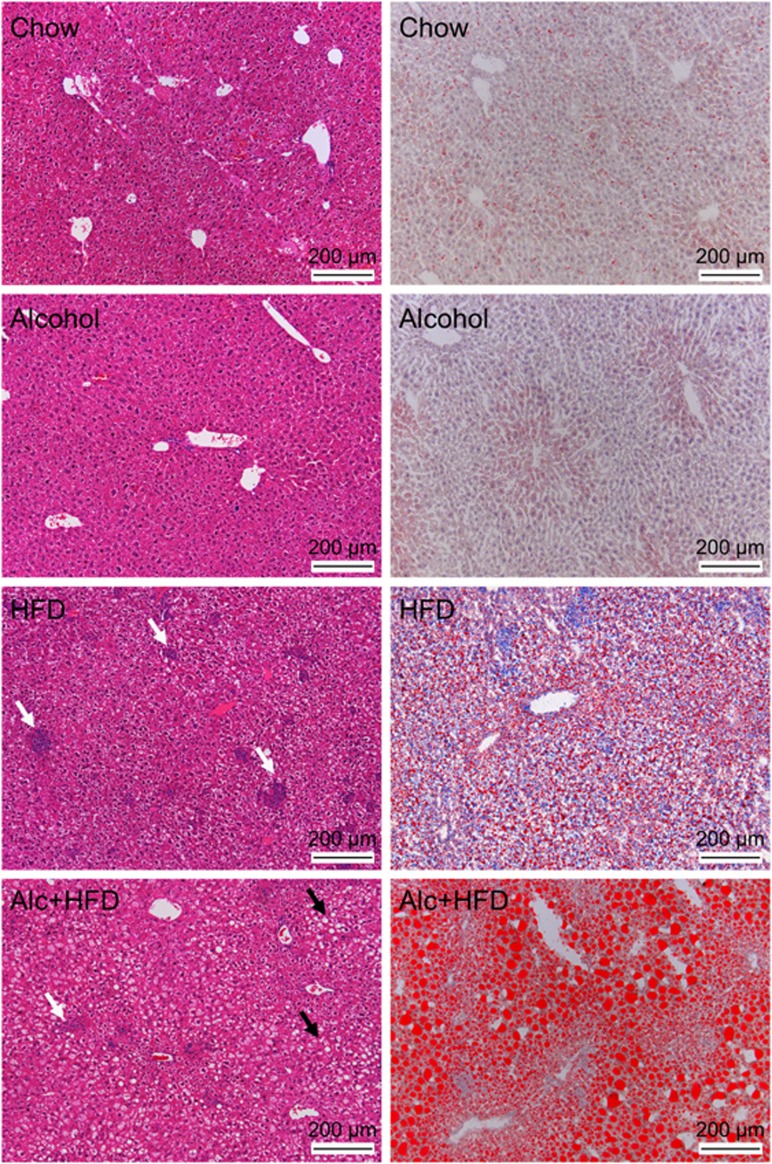
Alcohol and HFD increase lipid accumulation and cellular infiltration in mouse livers. *Left panel-H&E staining:* Maximum steatosis (black arrows) was observed in livers of Alc+HFD-treated mice and HFD-treated mice showed increased inflammatory cell clusters (white arrows) compared with control liver. *Right panel-ORO staining:* Maximum lipid accumulation (red) was observed in Alc+HFD-treated mice followed by HFD treatment. Alcohol only minimally increased steatosis. Representative images at × 10 magnification. H&E, haematoxylin and eosin; ORO, Oil Red O.

**Figure 3:**
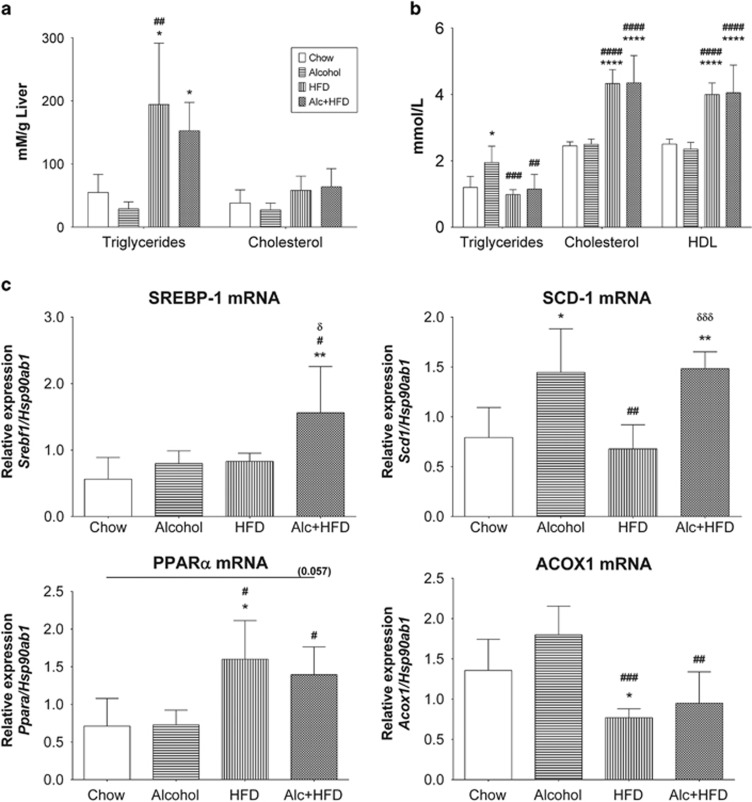
Alcohol and HFD induce hepatic steatosis. (**a**) HFD and Alc+HFD increased liver triglyceride. Analysis of liver homogenates showed that HFD- and Alc+HFD-treated mice had significantly higher triglyceride per gram of liver compared with livers from Chow- and Alcohol-treated mice (*n*=5). (**b**) Alcohol and HFD increased serum triglyceride, cholesterol and HDL. Serum triglyceride concentration was significantly higher in mice treated with Alcohol compared with control. It was significantly reduced with HFD and Alc+HFD when compared with Alcohol alone. Serum from mice treated with HFD and Alc+HFD showed significantly higher cholesterol and HDL than those on Chow diet (*n*=7–8). (**c**) Alcohol and HFD altered expressions of lipid processing mRNAs favouring lipid accumulation in the liver (Q-PCR). Alc+HFD induced significant expression of SREBP-1 mRNA compared with all other treatment groups. Alcohol alone and Alc+HFD induced significant expression of SCD-1 mRNA compared with Chow. SCD-1 expression was significantly reduced in HFD compared with Alcohol alone and Alc+HFD. mRNA expression of PPARα was significantly induced in HFD and Alc+HFD from both Chow and Alcohol treatment groups. HFD significantly reduced ACOX1 mRNA expression compared with Chow and Alcohol, while Alc+HFD significantly reduced ACOX1 expression compared with Alcohol. mRNA expression of target genes was normalised using housekeeper *Hsp90ab1* (*n*=5–6). Data are mean ±s.d. *P<0.05 from Chow, **P<0.01 from Chow, ****P<0.0001 from Chow; ^#^*P*<0.05 from Alcohol, ^##^*P*<0.01 from Alcohol, ^###^*P*<0.001 from Alcohol, ^####^*P*<0.0001 from Alcohol; ^δ^*P*<0.05 from HFD, ^δδδ^*P*<0.001 from HFD. HDL, high density lipoprotein; SREBP-1, sterol response element binding protein-1; SCD-1, stearoyl-CoA desaturase-1; Proliferation peroxisome activated receptor α, PPARα ACOX1, acyl-CoA oxidase 1.

Maximum steatosis was observed in Alcohol+HFD group compared with Chow and other treatments as shown by H&E staining (Figure 2, left panels) confirmed by ORO-stained increase in lipid droplets (red) in Alcohol+HFD-treated animals (Figure 2, right panels). Alcohol administration alone had minimal effect on steatosis. In contrast, the superimposition of alcohol on HFD induced both micro- and macro-vesicular lipid deposits in the livers of animals in the Alcohol+HFD group. Alcohol+HFD and HFD alone significantly increased triglycerides in the liver homogenates (Figure 3a) and reduced circulating triglycerides (Figure 3b) compared with Chow, validating the increased steatosis observed with H&E and ORO in these animals. Alcohol alone significantly increased serum but not liver triglyceride compared with Chow mice (Figures 3a and b). Liver LDL (mmol l^−^^1^) was considerably upregulated in HFD- (76±23) and Alcohol+HFD- (68±4) treated animals but could not be statistically compared with Chow and Alcohol due to below detection levels recorded for these groups. Moreover, circulating cholesterol and high density lipoprotein (HDL) also significantly increased with both Alcohol+HFD and HFD treatments compared with either Chow or Alcohol (Figure 3b).

We also examined the effects of treatments on the hepatic expression of genes related to lipid processing by Q-PCR. The *de novo* lipogenesis-related steroid response-element binding protein 1 (SREBP-1) mRNA expression was significantly increased by the combination of Alcohol+HFD compared with Chow and other treatment groups using one-way ANOVA (Figure 3c, SREBP-1). Alcohol alone and Alcohol+HFD significantly increased the stearoyl-CoA desaturase-1 (SCD-1) mRNA compared with the Chow and HFD groups (Figure 3c, SCD-1) showing primary effect was due to alcohol. A different pattern was observed for proliferation peroxisome activated receptor alpha (PPARα) mRNA expression, which was significantly increased with Alcohol+HFD and HFD alone (Figure 3c, PPARα). Conversely, the fatty acid β-oxidation gene Acyl-CoA oxidase 1 (ACOX1) mRNA expression was significantly reduced in the Alcohol+HFD and HFD alone interventions compared with the Alcohol alone or Chow fed animals (Figure 3c, ACOX1).

### Alcohol and HFD increased serum insulin

Serum insulin was increased above the Chow group (760±442 pg ml^−^^1^) with all treatments showing maximum and significant increase with Alcohol+HFD (3456±862 pg ml^−^^1^) followed by HFD (2768±646 pg ml^−^^1^), but did not reach significance with Alcohol (952±246 pg ml^−^^1^) (Table 3).

### Treatments increased cellular infiltration and expression of inflammation-related genes in mice livers inducing steatohepatitis

**Figure 4:**
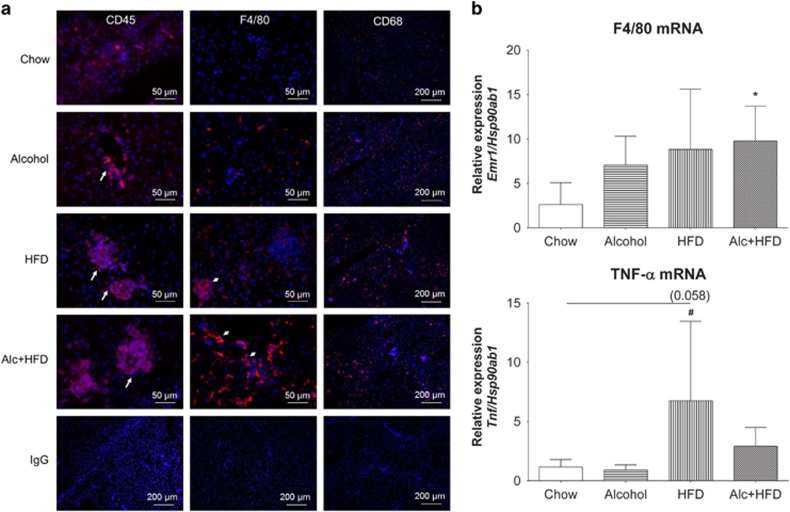
Alcohol and HFD increased hepatic inflammation. (**a**) Increase in number of leukocytes and macrophages in the livers of mice with treatments compared with Chow. Representative images of immunofluorescent stains. *CD45-positive leukocyte* clusters increased in number and size (arrows) in all groups compared with Chow. Both Alc+HFD and HFD treatment groups showed more CD45+ cells compared with Alcohol group. × 40 Magnification. *F4/80-positive Kupffer cells* increased in numbers with Alc+HFD, followed by HFD and Alcohol treatment compared with Chow. HFD treatment showed more large size clusters of F4/80-positive cells compared with Alc+HFD (small arrows). Alcohol alone did not show F4/80+ cell clustering. × 40 Magnification. *CD68-positive macrophages:* CD68+ cells increased with all treatments compared with Chow but did not show clustering of cells. × 10 Magnification. Isotype (IgG) controls (bottom panel) for specific antibodies did not show positive staining. × 10 Magnification. Nuclei were stained with DAPI (blue). (**b**) Alcohol and HFD increased expression of inflammation-related mRNAs (Q-PCR). All treatments induced expression of F4/80 mRNA compared with Chow, but only reached significance with Alc+HFD. HFD significantly induced TNF-α mRNA expression from Chow. Alc+HFD also increased TNF-α, but this was not significant, and Alcohol alone had no effect. mRNA expression of target genes was normalised using housekeeper *Hsp90ab1* (*n*= 5–6). Data are mean±s.d.; *P<0.05 compared with Chow; ^#^*P*<0.05 compared with Alcohol.

Cellular inflammatory infiltrate increased in all animals within each treatment group compared with Chow (Figure 4a). The number of CD45+ leukocytes increased with all treatments compared with Chow, with inflammatory cell clusters appearing in the Alcohol-treated mice and increasing in both cluster number and size in the HFD and Alcohol+HFD groups (Figure 4a, left panel). F4/80+ Kupffer cells also increased in number with all treatments compared to Chow with maximum numbers seen in Alcohol+HFD mice (Figure 4a, middle panel). Subset of CD68+ macrophages also increased with all treatments from Chow, with more intense staining in the HFD and Alcohol+HFD groups (Figure 4a, right panel). Quantitation of H&E-stained sections confirmed the total number of inflammatory cell clusters was significantly higher in the HFD and Alcohol+HFD groups (Table 4a). The numbers of large sized clusters (>15 cells per cluster) were significantly higher with HFD treatment followed by Alcohol+HFD (Table 4b; Figure 2, white arrows). In the Alcohol+HFD group, 75% (3 of 4) mice showed large inflammatory clusters compared with 63% (5 of 8) mice in the HFD group (Table 4b). Animals in the Alcohol group only had small to intermediate sized clusters.

Inflammation-related F4/80 mRNA expression was induced by all treatments compared with Chow, but only reached significance in the Alcohol+HFD group. The expression of tumour necrosis factor-alpha (TNF-α) was also induced with HFD and Alcohol+HFD treatments compared with Chow reaching significance for the animals treated with HFD (Figure 4b).

### Alcohol increased markers of fibrosis in the livers of HFD-treated mice

**Figure 5:**
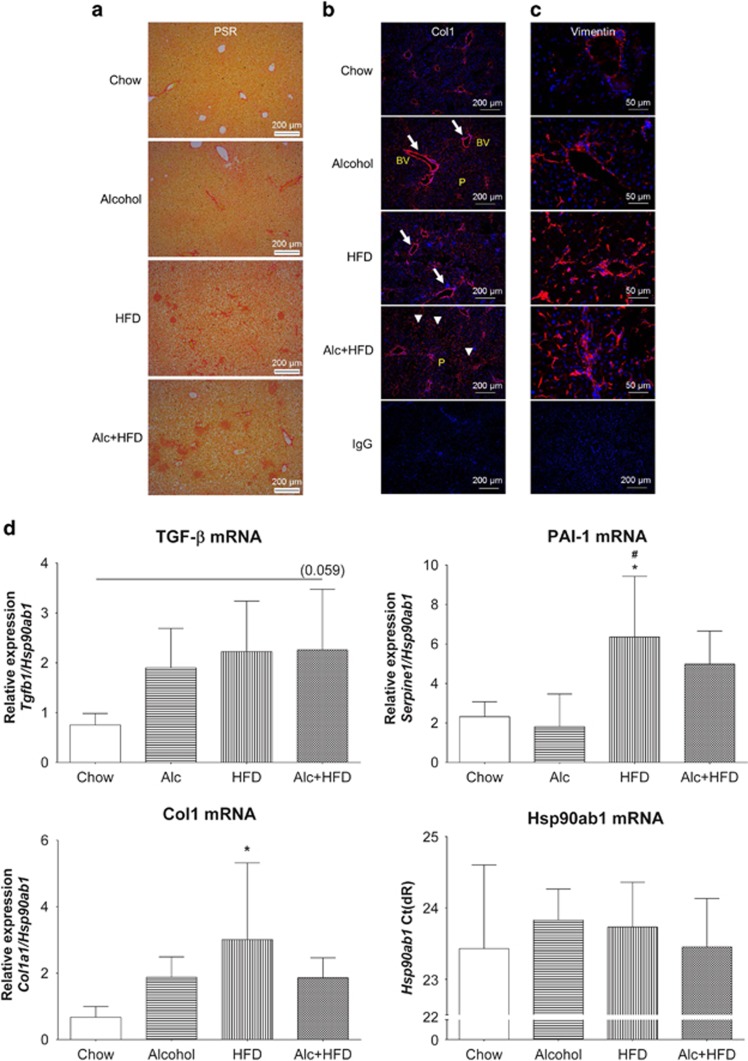
Alcohol and HFD induced hepatic fibrogenesis in mice. (**a**) Alcohol and HFD increased total collagen. PSR staining of paraffin-fixed liver sections showed increased collagen deposit in HFD- and Alc+HFD-treated mice compared with Chow. × 10 Magnification. (**b**) Alcohol and HFD increased Col1 (immunofluorescence). Immunofluorescent staining of frozen mouse liver tissues with anti-collagen 1 (Col1) antibody showed increasing collagen deposition (red) with all treatments around central and portal veins (arrows) with maximum staining visible in Alc+HFD mice livers across the lobule (arrowheads) compared with the other groups. Nuclei are stained with DAPI (blue). × 10 Magnification. (**c**) Alcohol and HFD increased vimentin-positive stellate cells in the liver. Vimentin-positive activated hepatic stellate cell (red) numbers increased with all treatments compared with Chow. Maximum increase was observed in HFD- and Alc+HFD-treated mice livers. Nuclei are stained with DAPI (blue). × 40 Magnification. IgG control at × 10 magnification. (**d**) Alcohol and HFD induced mRNAs of fibrosis-related molecules (Q-PCR). Expression of Col1 mRNA increased with all treatments compared with Chow but only HFD showed significance. TGF-β mRNA expression showed an increasing trend in all treatment groups compared with Chow but did not reach significance. PAI-1 mRNA expression increased significantly in HFD-treated mice compared with Chow and Alcohol groups. PAI-1 also increased with Alc+HFD but did not reach significance. mRNA expression of target genes was normalised using housekeeper *Hsp90ab1*. *Hsp90ab1* mRNA expression (Ct) did not change between treatment groups (*n*=5–6). Data are mean±s.d.; *P<0.05 from Chow; ^#^*P*<0.05 from Alcohol. BV, Blood vessel; P, Parenchyma; Ct, cycle threshold.

PSR staining showed increased collagen deposition in the livers of mice with all treatments compared with Chow. The most intense staining was seen in the Alcohol+HFD group (Figure 5a). Immunofluorescence staining with mouse-specific anti-Collagen 1 (Col1) antibody confirmed increased expression of Col1 (red) with treatments, specifically around the blood vessels (Figure 5b, arrows), showing maximum increase with Alcohol+HFD across the parenchymal lobule (arrowheads).

Activation of stellate cells was investigated by staining liver tissues for vimentin, another marker of fibrosis which is increased in myofibroblasts/hepatic stellate cells (HSCs). Increasing numbers of vimentin-positive stellate cells (red) were visible with all treatments compared with Chow and were most apparent in the Alcohol+HFD treatment group (Figure 5c). This increase was in line with the observed increase in collagen expression in this group.

Expression of the pro-fibrogenic markers TGF-β, Col1 and plasminogen activator inhibitor-1 (PAI-1) mRNAs was also increased by the various interventions. The greatest increase in TGF-β was observed in the Alcohol+HFD group, but this did not attain significance. HFD alone significantly upregulated Col1 and PAI-1 mRNAs compared with Chow, but this trend for PAI-1 increase did not reach significance in Alcohol+HFD-treated animals (Figure 5d).

## Discussion

This study has developed a clinically relevant and practical model that demonstrates recognised pathological features of the interactive effects of alcohol and HFD on the mouse liver. We have recapitulated the drinking patterns observed in the human setting of chronic intermittent alcohol consumption and westernised fat-rich diet to create a mouse model of steatohepatitis.

### Validity of the model: superimposing alcohol on HFD increases liver injury

In this work, we describe the 12-week Alcohol+HFD model in which causes of two of the most prevalent CLD, ALD and NAFLD have been combined to generate a more severe liver injury model of steatohepatitis. This model is useful to examine the effects and mechanisms of both diseases in a setting to systematically determine which pathogenic pathways are shared between both diseases, and which are specific for individual aetiology. The animals fed HFD superimposed with alcohol showed increased body weight, liver weight, worse metabolic profile with hyperlipidaemia and hyperinsulinaemia. Furthermore, progressive liver damage was characterised by elevated inflammation and more severe fibrosis with increased collagen deposition, associated activation of HSCs and TGF-β, over and above that with either agent alone, showing synergistic influence of alcohol and HFD and validating our model of more severe liver injury. Previously reported shorter models (2–4 weeks) of alcohol and HFD-induced steatohepatitis used more severe regimens of treatment observed clinically, using daily gavage with twice as much alcohol concentration as in the current model^13^ or alcohol concentrations reaching up to 32 g kg^−^^1^ body weight by intragastric infusion to induce inflammation.^14^ Both these models are harsh on animals, and intragastric infusion is technically quite complex, expensive and not widely available. By contrast, our longer term (12 weeks) model shows that a smaller dose given less frequently and with 48–96 h of recovery can also generate a useful model with similar features of steatohepatitis in addition to continuing weight gain and better welfare of animals and no observed mortality. In the current study, excessive handling was minimised and mice recovered within minutes of gavage at this alcohol dose. This lack of alcohol impact on normal animal behaviour, accessing food and water comparable to the saline group, may have protected the mice from weight loss described in the previous model.^13^ In addition, our model with intermittent but chronic alcohol binge is likely more relevant to the real life setting where people may drink 2–3 times a week, such as on weekends. As it is, this is a simpler model using gavage for alcohol administration that can be adopted in any animal laboratory. Even with 2 g kg^−^^1^ body weight of alcohol given twice a week, biochemical (cholesterol, triglyceride) and histopathological (H&E, ORO, F4/80, CD45, CD68) evidence of steatohepatitis was evident. Indeed, in this model, the HFD and alcohol regime also produced histological (PSR, Col1, vimentin) and molecular (TGF-β, Col1, PAI-1) profiles of fibrogenesis. This model may potentially be extended to produce more severe fibrosis by either increasing dose of alcohol or duration of treatment.

### Alcohol and HFD synergistically increase steatosis

HFD produced maximum changes in fat accumulation seen as micro-vesicular lipid droplets unlike alcohol alone. However, multiple doses of alcohol superimposed on HFD increased both micro- and macro-vesicular lipid deposits potentiating a synergistic injurious effect. This was confirmed at the molecular level with a significant increase in *de novo* lipogenic SREBP-1 and SCD-1 mRNAs expression coupled with a simultaneous decrease in β-oxidative ACOX1 mRNA indicating that the lipid processing pathway was directed towards lipid synthesis and away from catabolism in this combined setting of alcohol and HFD treatment. HFD alone and in combination with alcohol treatment induced transcription factor PPARα, suggesting upregulation of genes involved in lipid utilisation, lipid storage and insulin action.^17^ Insulin influences signalling between PPARα and SREBP1,^18^ and *de novo* fatty acids can further activate PPARα for lipid^19, 20^ and cholesterol homeostasis.^21^ We observed that the combined treatment of Alcohol+HFD synergistically increased lipid accumulation, insulin, triglyceride and cholesterol, underscoring the activation of PPARα pathway in this model. HFD alone primarily favoured the pathway for accumulation of triglyceride in the liver likely by reducing serum triglyceride, whereas Alcohol alone promoted the release of triglyceride in circulation.

### Alcohol and HFD synergistically increase inflammation

A major effect of the Alcohol+HFD treatment was increased inflammatory infiltration (F4/80+ Kupffer cells, CD45+, CD68+) in the livers of mice. The CD45+ leukocyte cell population not only increased, but the size of CD45+ cell clusters also increased especially with Alcohol+HFD. Both CD68+ and F4/80+ cells changed from an even spread across the parenchyma in Chow fed animals to a periportal distribution in Alcohol+HFD-treated livers. The increasing number of F4/80+ Kupffer cells correlated with increased F4/80 mRNA expression, specifically in Alcohol+HFD animals. This pattern was different for TNF-α mRNA where the increased expression mainly resulted from the HFD. These changes, however, were not reflected in liver enzymes (ALT, AST) because the blood samples obtained were much later than the optimal peak period of 9 h post alcohol administration.^11^ Nonetheless, this model demonstrated histological and molecular features characteristic of established steatohepatitis with increased steatosis and an increased inflammatory response after exposure to the combination of alcohol and HFD.

### Alcohol and HFD synergistically increase histological and molecular profile of pro-fibrogenesis

In our model of Alcohol+HFD, increased vimentin-positive cells along the sinusoidal areas throughout the parenchyma suggest activation and proliferation of HSCs/myofibroblast-like cells, typical characteristics of initiation of fibrosis during liver injruy. Additionally, this increase in HSCs was associated with increased collagen deposition as shown by PSR and Col1 staining providing further evidence of a pro-fibrogenic response in the livers of Alcohol+HFD-treated animals. Furthermore, increase in the molecular indicators of fibrogenesis such as TGF-β, Col1 and PAI-1 indicates a pro-fibrogenic profile in this model. The increase in the expression of these markers was not synergistic as HFD primarily increased Col1 and PAI-1 mRNA and the most evident influence of Alcohol+HFD was mainly on TGF-β (Table 5). In addition, the patterns of change were more obvious at the protein level suggesting that the expression of extracellular matrix turnover markers may also be altered.

In conclusion, superimposing alcohol on the HFD induced obesity and exacerbated the extent of liver damage caused by either agent alone in our model. HFD alone induced several measures of liver injury, whereas the main effect of alcohol was seen as an increase in liver weight, serum triglyceride and lipogenic SCD-1 mRNA. Alcohol also exerted additional effect over and above HFD by increasing micro- and macro-vesicular lipid deposits, SREBP-1, inflammatory Kupffer cells, and fibrogenic HSCs and TGF-β. This model utilising two of the most common risk factors for liver disease develops steatohepatitis and shows evidence of pro-fibrogenic changes. We have created a physiologically appropriate model that is easy to establish in short term and is relevant to study the development of fibrosis.

## Figures and Tables

**Table 1:** Treatments had no effect on kidney-, heart-, spleen-to-body weight

	*Chow*	*Alcohol*	*HFD*	*Alcohol+HFD*
Kidney	1.00±0.18	1.13±0.05	1.11±0.09	1.03±0.14
Heart	1.00±0.16	0.93±0.09	0.98±0.13	1.01±0.19
Spleen	1.00±0.13	1.11±0.35	1.31±0.45	1.11±0.25

Abbreviation: HFD, high fat diet. No significant change in weight (fold-change from Chow) was observed for kidney, heart and spleen with treatments (*n*=7–8). Data are mean±s.d.

**Table 2:** Liver enzymes were not affected by treatments

	*ALT (U l*^−^^1^*)*	*AST (U l*^−^^1^*)*	*AST:ALT*	*ALP (U l*^−^^1^*)*
Chow	45.0±31.69	104.5±32.05	2.93±1.14	75.0±3.79
Alcohol	36.4±16.10	72.9±16.12*	2.15±0.57	61.7±10.23
HFD	38.1±20.61	88.3±31.41	2.56±0.78	48.4±12.42**
Alcohol+HFD	34.5±13.25	91.5±10.69	2.96±1.07	55.5±6.5***

Abbreviations: ALP, alkaline phosphatase; ALT, alanine aminotransferase; AST, aspartate transaminase; HFD, high fat diet. No significant change in liver enzymes (ALT and AST:ALT) was observed with any treatment, except AST was significantly reduced in Alcohol alone from Chow. A significant decrease was observed for ALP with all treatments compared with Chow (*n*=7–8). Data are mean±s.d.; *P⩽0.05 from Chow; **P⩽0.01; ***P⩽0.001.

**Table 3:** Circulating insulin (pg ml^−^^1^) increased with HFD and Alc+HFD

*Treatment*	*Insulin (pg ml*^−^^1^*)*
Chow	764.6±369.2
Alcohol	958.6±159.2
HFD	2780±572.0^a^
Alcohol+HFD	3362±867.4^a^^,^^b^

Abbreviations: Alc, alcohol; HFD, high fat diet. Circulating insulin significantly increased in mice treated with HFD and Alc+HFD (*n*=7–8). Data are mean±s.d.

a *P*<0.0001 compared with Chow.

b *P*<0.0001 compared with Alcohol.

**Table 4a:** Treatments induced inflammatory cell clusters in mice livers. Total number of inflammatory infiltrate clusters increased in every mouse in all treatment groups compared with Chow

*Treatment*	*No. of mice*		*Total no. of clusters (mean±s.d.)*
	*No clusters (0 cells)*	*Clusters (>5 cells)*	
Chow (*n*=4)	4	0	0.0±0.0
Alcohol (*n*=4)	0	4	5.750±3.500
HFD (*n*=8)	0	8	12.75±5.800**
Alc+HFD (*n*=4)	0	4	11.75±5.500*

Abbreviations: Alc, alcohol; HFD, high fat diet; χ^2^ analysis and Fisher's exact test comparing numbers of mice with or without clusters. Ordinary one-way ANOVA with Tukey's multiple comparisons test; *P<0.05, **P<0.01.

**Table 4b:** Treatments induced inflammatory cell clusters in mice livers. HFD and Alc+HFD treatments significantly induced large inflammatory cell clusters compared with Chow

*Treatment*	*No. of mice*			*Large clusters (>15 cells)*
	*No clusters (0 cells)*	*Small clusters (1–5 cells)*	*Intermediate clusters (6–15 cells)*	
Chow (*n*=4)	4	0	0	0
Alcohol (*n*=4)	0	2	2	0
HFD (*n*=8)	0	1	2	5**
Alc+HFD (*n*=4)	0	1	0	3*

Abbreviations: Alc, alcohol; HFD, high fat diet. χ^2^ analysis and Fisher's exact test comparing numbers of mice with or without clusters. Ordinary one-way ANOVA with Tukey's multiple comparisons test; *P<0.05, **P<0.01.

**Table 5:**
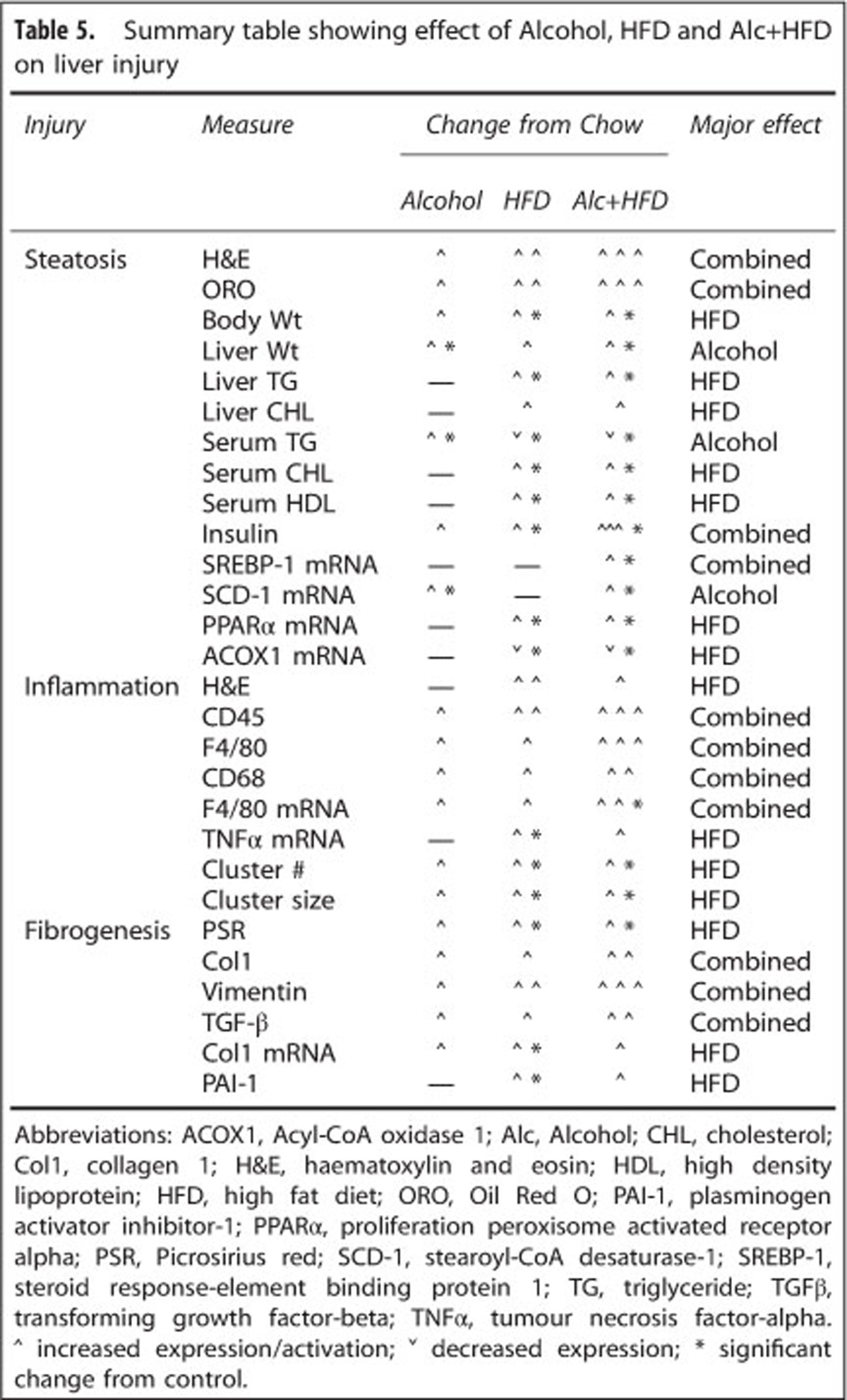
Summary table showing effect of Alcohol, HFD and Alc+HFD on liver injury
