# Supplementary material for: Effect of multiple binge alcohol on diet-induced liver injury in a mouse model of obesity
Source: Nutr Diabetes. 2015 Apr 27;5(4):e154–. doi: 10.1038/nutd.2015.4 (PMC4423200; doi:10.1038/nutd.2015.4)
Supplement: Supplementary Tables 1-3 [file nutd20154x1.docx]

**Supplementary Table 1. Amount and volume of ethanol per gram (g) body weight per mouse for 2 g/kg dose as 30% solution in saline.**

| **Body weight (g)** | **20** | **21** | **22** | **23** | **24** | **25** | **26** | **27** | **28** | **29** | **30** | **31** | **32** |
| --- | --- | --- | --- | --- | --- | --- | --- | --- | --- | --- | --- | --- | --- |
| **Amount EtOH required (mg)** | 40 | 42 | 44 | 46 | 48 | 50 | 52 | 54 | 56 | 58 | 60 | 62 | 64 |
| **Volume of EtOH (μL)** | 50.63 | 53.16 | 55.70 | 58.23 | 60.76 | 63.29 | 65.82 | 68.35 | 70.89 | 73.42 | 75.95 | 78.48 | 81.01 |
| **Amount EtOH as 30% total vol (μL)** | 133 | 140 | 147 | 153 | 160 | 167 | 173 | 180 | 187 | 193 | 200 | 207 | 213 |

| **Body weight (g)** | **33** | **34** | **35** | **36** | **37** | **38** | **39** | **40** | **41** | **42** | **43** | **44** | **45** |
| --- | --- | --- | --- | --- | --- | --- | --- | --- | --- | --- | --- | --- | --- |
| **Amount EtOH required (mg)** | 66 | 68 | 70 | 72 | 74 | 76 | 78 | 80 | 82 | 84 | 86 | 88 | 90 |
| **Volume of EtOH (μL)** | 83.54 | 86.08 | 88.61 | 91.14 | 93.67 | 96.20 | 98.73 | 101.27 | 103.80 | 106.33 | 108.86 | 111.39 | 113.92 |
| **Amount EtOH as 30% total vol (μL)** | 220 | 227 | 233 | 240 | 247 | 253 | 260 | 267 | 273 | 280 | 287 | 293 | 300 |

Alcohol mice were treated with a 30% ethanol (EtOH) solution such that they would receive 2 g of ethanol per kilogram per gavage twice a week for 12 weeks. Saline mice were treated with saline such that they would receive saline volume equivalent to 2 g/kg gavage twice a week for 12 weeks. There were no notable differences between the HFD and the normal diet.

**Supplementary Table 2. Observations on mice welfare and behaviour over time following treatment.**

| **Clinical Examination** | **Abnormal**  **signs** | **Week 1** | | **Week 4** | | **Week 8** | | **Week 12** | |
| --- | --- | --- | --- | --- | --- | --- | --- | --- | --- |
|  |  | Saline | Alcohol | Saline | Alcohol | Saline | Alcohol | Saline | Alcohol |
| **Active** | Passed out | no | no | no | no | no | no | no | no |
|  | Uncoordinated | no | no | no | 5 to 10 min | no | 15 to 30 min | no | 30 min to 1 hr |
|  | Drowsy | no | no | no | 5 to 10 min | no | 15 to 30 min | no | no |
| **Activity (Provoked and unprovoked)** | Reduced (relative to saline treated mice) | no | no | no | 5 to 10 min | no | 5 to 10 min | no | no |
| **Normal eating and drinking** | Not eating | Resumed after 10 min | Resumed after 10 min | Resumed after 10 min | Resumed after 20 min | Resumed after 10 min | Resumed after 20 min | Resumed after 10 min | Resumed after 20 min |

Mice were monitored extensively following each dose of alcohol or saline every 30 minutes for two hours, then every hour (up to six hours total) to ensure full recovery. Initially no observable behavioural changes were seen in the mice given a 2 g/kg dose of alcohol. The alcohol did not appear to have an effect until week three. From week four, mice administered alcohol were slow to react, drowsy and had uncoordinated movements compared to those given saline, for up to 10 minutes.

**Supplementary Table 3. Antibodies for Immunofluorescence and Primers for qPCR (alphabetical)**

| **Primary antibodies (specific for *mus musculus*)** | | | | | | | | | | | |
| --- | --- | --- | --- | --- | --- | --- | --- | --- | --- | --- | --- |
| **Marker** | | **Host** | | **Clone (Isotype)** | **Dilution** | | **Catalogue ID** | **Supplier/ Reference** | | | |
| CD45 antigen (CD45) | | Rat | | 30-F11 (IgG_2b_, κ) | 1:50 | | 550539 | BD Bioscience, San Jose, CA, USA | | | |
| CD68 antigen (CD68) | | Rat | | FA-11 (IgG_2a_) | 1:100 | | MCA1957BT | AbD SeroTec, Kidlington, Oxford, UK | | | |
| Collagen, type 1 (Col1) | | Rabbit | | IgG | 1:50 | | ab34710 | Abcam, Cambridge, UK | | | |
| F4/80 glycoprotein antigen (F4/80) | | Rat | | IgG_2b_ | 1:2 | | N/A | {Austyn, 1981 #826} | | | |
| Vimentin (VIM) | | Rat | | 280618 (IgG_2a_) | 1:200 | | MAB2105 | R&D systems, Minneapolis, MN, USA | | | |
| **Secondary antibodies** | | | | | | | | | | | |
| **Specificity** | | **Host** | | **Conjugated fluorophore** | **Dilution** | | **Catalogue ID** | **Supplier** | | | |
| Rat | | Donkey | | Alexa Fluor® 594 | 1:400 | | A-21209 | Life Technologies Australia Pty Ltd, Mulgrave, VIC, Australia | | | |
| Rabbit | | Donkey | | Alexa Fluor® 594 | 1:400 | | A-21207 |  |  |  |  |
| **Primers for qPCR** | | | | | | | | | | | |
| **Primer set** | **Gene name** | | **Forward (5’ 🡪 3’)** | | | **Reverse (5’ 🡪 3’)** | | | **Product size (bp)** | **T_anneal_ (°C)** | **Reference/ Accession number** |
| *Acox1* | Acyl-Coenzyme A oxidase 1, palmitoyl (ACOX1) | | GCCCAACTGTGACTTCCATC | | | GCCAGGACTATCGCATGATT | | | 73 | 60 | NM_015729.3 |
| *Col1a1* | Collagen, type I, alpha 1 (Col1) | | GAGCGGAGAGTACTGGATCG | | | GCTTCTTTTCCTTGGGGTTC | | | 158 | 55 | {Syn, 2009 #981@@author-year} |
| *Emr1* | F4/80 glycoprotein antigen (F4/80) | | CTTTGGCTATGGGCTTCCAGTC | | | GCAAGGAGGACAGAGTTTATCGTG | | | 165 | 60 | NM_010130.4 |
| *Hsp90ab1* | Heat shock protein 90 alpha (cytosolic), class B member 1 | | CTCGGCTTTCCCGTCAAGAT | | | GTCCAGGGCATCTGAAGCAT | | | 174 | 55 | NM_008302.3 |
| *Ppara* | Peroxisome Proliferation-activated receptor alpha (PPARα) | | TCTGGAAGCTTTGGTTTTGC | | | TTCGACACTCGATGTTCAGG | | | 175 | 55 | {Sharara-Chami, 2012 #878@@author-year} |
| *Scd1* | Stearoyl-Coenzyme A desaturase 1 (SCD-1) | | TTCCCTCCTGCAAGCTCTAC | | | CAGAGCGCTGGTCATGTAGT | | | 62 | 60 | NM_009127.4 |
| *Serpine1* | Plasminogen activator inhibitor type 1 (PAI-1) | | ATGCCATCTTTGTCCAGCGG | | | TTGGTATGCCTTTCCACCCAG | | | 151 | 55 | {Seth, 2008 #317@@author-year} |
| *Srebf1* | Sterol regulatory element binding protein-1 (SREBP-1) | | CAGCTCAGAGCCGTGGTGA | | | TTGATAGAAGACCGGTAGCGC | | | 70 | 60 | NM_011480.3 |
| *Tgfb1* | Transforming growth factor, beta 1 (TGF-β) | | CCTTCCTGCTCCTCATGGCCA | | | GTCCTTCCTAAAGTCAATGTA | | | 149 | 50 | {Sahai, 2004 #524@@author-year} |
| *Tnf* | Tumour necrosis factor alpha (TNF-α) | | CAGCCTCTTCTCATTCCTGCTTG | | | GGGTCTGGGCCATAGAACTGA | | | 134 | 55 | NM_013693.3 |
